# Exploring textile-based electrode materials for electromyography smart garments

**DOI:** 10.1177/20556683211061995

**Published:** 2022-02-01

**Authors:** Emily Lam, Milad Alizadeh-Meghrazi, Alessandra Schlums, Ladan Eskandarian, Amin Mahnam, Bastien Moineau, Milos R Popovic

**Affiliations:** 1 Institute of Biomedical Engineering, 7938 University of Toronto, Toronto, Canada; 2 Myant Inc, Etobicoke, Canada; 3 The KITE Research Institute, 153174 Toronto Rehabilitation Institute – University Health Network, Toronto, Canada; 4 Department of Mechanical and Mechatronics Engineering, 120492 University of Waterloo, Waterloo, Canada; 5 Department of Materials Science and Engineering, 113479 University of Toronto, Toronto, Canada

**Keywords:** Bioelectric data acquisition, biomaterials electromyography (EMG), fabric, monitoring, prosthetic control

## Abstract

**Introduction:**

In recent years, electromyography (EMG) has been increasingly studied for wearable applications. Conventional gel electrodes for electrophysiological recordings have limited use in everyday applications such as prosthetic control or muscular therapy at home. This study investigates the efficacy and feasibility of dry-contact electrode materials employed in smart textiles for EMG recordings.

**Methods:**

Dry-contact electrode materials were selected and implemented on textile substrates. Using these electrodes, EMG was recorded from the forearm of able-bodied subjects. 25% and 50% isometric maximum voluntary contractions were captured. A comparative investigation was performed against gel electrodes, assessing the effect of material properties on signal fidelity and strength compared.

**Results:**

When isolating for electrode surface area and pressure, 31 of the 40 materials demonstrated strong positive correlations in their mean PSD with gel electrodes (r > 95, *p* < 0.001). The inclusion of ionic liquids in the material composition, and using raised or flat electrodes, did not demonstrate a significant effect in signal quality.

**Conclusions:**

For EMG dry-contact electrodes, comparing the performance against gel electrodes for the application with the selected material is important. Other factors recommended to be studied are electrodes’ durability and long-term stability.

## Introduction

The growing popularity of wearable devices has generated a great deal of clinical potential for continuous monitoring of electrophysiological or biopotential signals. These signals contain a wealth of data that can be used to detect, diagnose, monitor, treat, and manage chronic and acute conditions. Specifically, electromyography (EMG) can be used to record the underlying electrical activity of an individual’s muscles. Long-term EMG monitoring could be used by an amputee or paraplegic to control their prosthesis or interact with rehabilitation devices.^1^ Alternatively, unobtrusive EMG monitoring could help athletes optimize training and performance.^2^ The broad interest and wide range of potential applications for pervasive wearable devices for continuous electrophysiological measurements requires devices that mitigate the barriers to adoption, such as expensive, bulky, and uncomfortable form factors.

The functionalization of textiles via the use of conductive materials, better known as smart textiles, presents an opportunity for improvement in continuous electrophysiological monitoring. Textiles are ubiquitous and introduce little habit change, making them an attractive medium for technology integration. Smart textiles are well-suited to scalable mass manufacturing as they employ established textile fabrication techniques such as knitting,^3^ weaving,^4^ and embroidery.^5^ This makes the manufacturing of smart textiles simple, scalable, and highly customizable. Functionalization of garments can also be introduced by screen printing,^6–8^ inkjet printing,^9–11^ or coating^12–14^ conductive layers onto textile substrates.

In continuous electrophysiological monitoring, the electrodes form a critical interface with the body known as the electrodeskin interface. The outermost layer of the skin, the stratum corneum (SC) contains dried layers of keratin, giving it a high impedance and causing it to act as a barrier to biopotential signals.^15^ Electrodeskin impedance can be thought of as a measure of how effectively electric charges might be transferred from the electrode to the skin.^16^ Electrodes with higher impedance are more susceptible to noise from electrical interference^17^ and motion artifacts.^18^ To minimize noise, it is recommended to minimize electrodeskin impedance to lower impedance mismatch between electrodes.^15,19,20^

The gold-standard biopotential electrodes are “wet electrodes,” which use an electrolytic hydrogel as a conduit for charge transfer between the skin and the electrode. Wet electrodes overcome the high impedance of the SC by hydrating it, effectively easing the transfer of biopotentials. While hydrogel electrodes provide high-quality signal, the gel and adhesive needed to keep the electrode attached to the skin can cause irritation and allergic reactions.^21^ Wet electrodes also dry out, resulting in the degradation of signal quality over time.

Textile electrodes are “dry” and do not use an electrolyte, making them well-suited for long-term and day-to-day use. While using dry electrodes eliminates the issue of electrode dehydration, the lack of an electrolyte can lead to interface impedances up to three orders of magnitude greater than those achieved with the gel electrodes.^20^ With smaller amplitude signals and increased sensitivity to motion artifact and electromagnetic interferences, dry electrodes typically exhibit poorer signal quality.^21–23^ However, material choice can have a profound impact on dry electrode signal quality.

Researchers have investigated a variety of materials including silver,^3,5,6,8,22,24^ carbon rubber,^7,25^ graphene,^12,13,26^ carbon black,^27–29^ carbon nanotubes (CNT),^30,31^ and poly (3,4-ethylenedioxythiophene) doped with poly (styrenesulfonate) (PEDOT:PSS).^6,9–11,14^ While these advances in developing dry electrodes are valuable, the current literature does not provide much guidance in comparing these materials. Reviews have been published focusing on materials for dry electrodes and their applications,^21,32,33^ but have not compared the materials’ ability to capture biopotential signals. Most studies involving dry-textile electrodes have focused on electrocardiography (ECG), which have much stronger signal amplitudes compared to EMG. Additionally, many of the papers focus on introducing new materials and only test one material at a time, typically in comparison to a hydrogel electrode, sometimes without standardized metrics for evaluation. Castrillón et al. performed a comparative study of PEDOT:PSS-treated fabrics to understand their feasibility for ECG measurement.^34^ The research done by Castrillón et al. is a helpful synthesis of the literature, and this work aims to achieve a similar goal for EMG.

To understand how novel electrode materials compare, this work compares existing textile and textile-based electrode materials in terms of signal-to-noise ratio (SNR). SNR is an indicator of signal quality, defined as the ratio of energy in the EMG signal to the energy in the noise, or undesirable, part of the signal.^35^ This paper provides an overview of the current state of the art in textile and textile-based electrodes to inform the materials tested. Materials of interest were fabricated using knitting and screen printing, tested with an agar model to characterize impedance, and used to measure EMG signals. This extensive comparison of electrode materials aims to inform future development of smart textile solutions for electrophysiological monitoring by establishing which materials should be further developed and tested.

## Dry biopotential electrode materials: Current status

*Conductive yarns* come in many forms and can be developed from ferrous alloys, nickel, nickel alloys, stainless steel, titanium, aluminum, copper, silver, carbon, and carbon rubber.^36^ Silver boasts high conductivity and easy manufacturability, but has poor washability, low resistance to mechanical strain, and is susceptible to oxidation.^37^ Often times, silver electrodes are moistened to provide better signal quality.^5,24^ In search of improved signal quality, researchers have investigated other materials, especially conductive polymers, carbon derivatives, and nanomaterials. A brief survey of these is reported in Table 1.

**Table 1.** Dry electrode materials, impedances, and signal quality.

Material	Size (cm^2^)	Skin prep	Interface pressure	Impedance	Signal quality and application
Sewn Ag-plated thread^24^	1, 2, 4, 8, or 16^A^	N.R.	Pressure from band	N.R.	*ECG:* Able to distinguish R-R intervals
Woven Ag into shorts^4^	39 or 42	Skin shaved, abraded, and cleansed	Pressure from shorts	> 10kΩ	*EMG (knee extension):* Bland-Altman plots showing good agreement (<2SD) between textile and hydrogel electrodes
Knitted Ag^3^	N.R.	None, but used electrode gel and saline solution	Pressure from headband	N.R.	*EEG:* Strong correlation (R^2^ > 0.7) for most frequencies with both gel and saline electrolytes
Embroidered Ag and Ag/Ti threads^5^	14	None, but electrodes were moistened with water	Pressure from band	N.R.	*ECG:* Suitable for heart rate variability measurement at rest and in motion when wet
Screen-printed Ag on textile^6^	∼11.5	N.R.	Adjustable belt	* 2MΩ-< 500 kΩ from 0.1-200 Hz	*ECG:* SNR = 20.8–22.4dB PP_amp_ = 124.8–330.2 µV
Screen-printed Ag/AgCl on textile^8^	∼4.9	N.R.	∼12 mmHg	* 10–100 kΩ from 0.1Hz1 kHz	*ECG:* SNR = 26.68dB SNR_H_ = 26.70 dB
Ag nanowires in PDMS^22^	N.R.	Cleaned with 70% isopropyl alcohol	∼14 mmHg	* ∼5MΩ-<1MΩ from 40Hz-100 kHz	*ECG:* Able to distinguish P-wave, QRS-complex, and T-wave *EMG (extensor digitorum communis):* SNR = 24.7 dB SNR_H_ = 27.3 dB MNF = 115.2 Hz MNF_H_ = 119.1 Hz MDF = 135.6 Hz MDF_H_ = 139.0 Hz
Ethylene propylene diene monomer with ∼50% carbon^25^	N.R.	N.R	Pressure from adhesive	+ ∼100 Ω/cm^2^ at 10 Hz Δ ∼1MΩ/cm^2^ at 10 Hz * 1– 10MΩ/cm^2^ at 10 Hz	*ECG:* R^2^= 0.99 between collected signals, R-peak and T-waves detectable *EEG*: R^2^ > 0.7 (strong)
Screen-printed carbon-loaded rubber on textile^7^	∼0.8	N.R.	Pressure from headband	∇ ∼1000-<100 Ω from 20Hz−10 MHz at 25 kPa	*EMG (jaw clench):* RMS = 0.157–0.162 *EOG (eyebrow raise):* RMS = 0.437–1.12
Graphene-coated electrode^12^	∼4.5 or ∼11.3	N.R	Pressure from adhesive	* 445.05−13.82 kΩ from 20 to 1000 Hz	*ECG:* SNR = 25.32dB SNR_H_ = 21.23 dB
rGOx-coated nylon^26^	∼6	N.R.	Pressure from adhesive	* 50.9−2.2 kΩ from 10 to 1000 Hz	*ECG:* 97% cross-correlation with simultaneously collected ECG from hydrogel electrodes
CB/PDMS^27–29^	∼3.1 or ∼7.1	*ECG:* Cleaned with 70% isopropyl alcohol *EMG:* Wiped with 2% alcohol pad	Elastic compression bandage (“low,” “medium,” and “high”)	* 274–378 kΩ at 33 Hz, “medium” pressure	*ECG:* R^2^ > 0.9 for all tested conditions *EMG (biceps brachii, triceps brachii, tibialis anterior):* Tested underwater, RMS for all muscles showed R^2^ > 0.7 between electrode
Multi-wall CNT/PDMS^31^	∼3.1	None	Compression bandage	* ∼10MΩ at 1 Hz	*ECG:* P-wave, QRS-complex, and T-wave clearly distinguished
Multi-wall CNT/PDMS with micropillars^30^	∼0.8^B^	N.R.	Pressure from adhesive	348kΩ at 30 Hz	*ECG:* P-wave, QRS-complex, and T-wave clearly distinguished
Screen-printed PEDOT:PSS on textile^6^	∼11.5	N.R.	Adjustable belt	* > 2MΩ-< 500kΩ from 0.1-200 Hz	*ECG:* SNR =20.1–20.2dB PP_amp_ = 143.8–363.9 µV
Inkjet-printed PEDOT:PSS on paper^9^	∼0.8	N.R.	N.R.	* 1.171.40 MΩ at 1 Hz	*ECG:* SNR = 10.28–11.01 dB
Raised PEDOT:PSS dyed polyester^14^	9	N.R.	Chest belt	* 402.5 kΩ from 20 to 500 Hz	*ECG:* S = > 90%, and P = > 85% at rest, S = < 70%. and P = < 70% when walking
Etched and inkjet-printed PEDOT:PSS electrodes on Au with IL gel^10,11^	∼0.5	N.R.	Pressure from adhesive	* ∼10MΩ-<10kΩ from 0.1Hz to 10 kHz	*ECG:* SNR_dry_ = 12.93dB SNR_IL_ = 13.75 dB

Ag: silver; SD: standard deviation; PDMS: polydimethylsiloxane; Ti: titanium; AgCl: silver chloride; rGOx: reduced graphene oxide; CB: carbon black; CNT: carbon nanotube; PEDOT:PSS: poly(3,4-ethylenedioxythiophene) polystyrene sulfonate; Au: gold; IL: ionic liquid; N.R.: not reported; H: hydrogel (Ag/AgCl); ECG: electrocardiogram; EMG: electromyogram; EEG: electroencephalogram; SNR: signal-to-noise ratio; PP_Amp_: peak-to-peak amplitude; MNF: mean frequency; MDF: median frequency; CV: coefficient of variation (%); RMS: root mean square; S: sensitivity (%); P = predictivity (%); *Skinelectrode impedance tested on human participants; +Electrode impedance tested on metal plates; ΔElectrode impedance tested in a skin phantom; ∇Electrodes placed face to face; ^A^Total surface area of all electrodes, not size per electrode; and ^B^Not including the surface area of the micropillars.

*Carbon derivatives* boast a combination of electrical and ionic conductance, generally having a resistance much higher than silver. This higher resistance can act as a filter, reducing the amplitude of the biopotential recorded and making the electrode less sensitive to noise.^20^ Carbon-loaded rubbers have also been used to create bulk electrodes using stencil-printing or curing in a mold.^25,38^

*Graphene* boasts high mechanical strength, good electrical and thermal conductivity, a large specific surface area, and chemical stability.^21^ Graphene has been coated on top of commercial ECG electrodes.^12^ Electrodes utilizing easier-to-process, hydrophilic graphene materials, such as reduced graphene oxide (rGOx) have also been constructed.^13,26^

*Carbon black (CB)* has high surface area and degree of porosity, allowing it to have electrical conductivity even under low loading within polymer composites.^39^ Additionally, CB can improve fracture, abrasion, and failure properties; coating with a CB polymer could help preserve the flexibility of textile materials.^40^ Biocompatibility testing with CB/polydimethylsiloxane (PDMS) electrodes has found that CB/PDMS is not cytotoxic to connective tissue cells and primary human epidermal keratinocytes.^27–29^

*Carbon nanotubes (CNTs)* have high mechanical strength, good electrical conductivity, and can be produced at a relatively low cost.^21^ The biocompatibility of CNT/PDMS electrodes has been evaluated in vitro by culturing human epithelial cells on the electrodes and through continuous wear over the course of a week, where no skin reactions (i.e. itching or erythema) were reported.^41^

*Poly(3,4-ethylenedioxythiophene) polystyrene sulfonate (PEDOT:PSS)* has a stable conducting state, biocompatibility, and easy processability.^42^ PEDOT:PSS electrodes have been constructed using screen printing,^6^ inkjet printing,^9^ and dyeing.^14,34^

*Ionic liquids (ILs)* consist of ions with melting points or glass-transition temperatures below 100°C, such that they are liquid at room temperature.^43^ ILs have been polymerized into gels to reduce skinelectrode impedance in biopotential electrodes.^10,11^

*Raised electrodes* are textile electrodes containing a foam or stuffing, creating a raised geometry.^3,8,14^ This shape is thought to improve electrode contact by increasing and improving uniformity of pressure at the electrodeskin impedance. Improved contact allows the electrode to conform better to the shape of the skin, thereby increasing the interface area and reducing electrodeskin impedance.^21,22^ This can improve signal quality, even during movement.^8^

## Materials and methods

This study investigates a series of textile-based electrodes for the measurement of EMG. They were compared with gold-standard hydrogel electrodes (Kendall, Covidien) in their signal-to-noise ratio (SNR) and power spectral density (PSD). The study was organized into three phases to minimize the number of materials that needed to be tested with participants. The phases were (1) electrode preparation, (2) impedance testing, and (3) EMG testing on participants.

### Electrode preparation

Based on a review of the literature, materials appropriate for simple commercial fabrication of textile-based sensors were gathered for testing (Table 2). The process flow detailing material selection for the electrodes in this study can be found in a prior study by the same team, assessing the electrode properties on an agar skin model.^44^ Knitted electrodes were tested with and without screen-printed polymer coating. Knitted electrodes were produced using an 18-gauge flatbed knitting machine (CMS-ADF, Stoll, Germany) using either 200 denier silver or 640 denier carbon yarn. The yarns consist of nylon 6,6 filaments with a round cross-section with electrically conductive silver plating or suffused carbon particles on the surface. The resistance of the silver yarn was 112.3 Ω/m, while the resistance of the carbon yarn was too high to be measured by the multimeter (Fluke 8845 A, Fluke Corporation, United States), which had a limit of 20 M Ω.

**Table 2.** Screen-printed coatings.

Label	Coating condition^a^
0	None
1	Carbon
2	Carbon + 5% IL
3	XCMB + 10% graphene
4	XCMB + 10% graphene + 5% IL
5	Neoprene + CB (7%)
6	Neoprene + CB (7%) + 5% IL
7	PEDOT:PSS +12.5% (PDMS + 1% CNT)
8	PEDOT:PSS + 12.5% (PDMS + 1% CNT) + 5% IL
9	PDMS + 1% CNT
10	PDMS + 1% CNT + 5% IL

IL: ionic liquid; XCMB: 1-ethyl-3-methylimidazolium bis(triflouromethylsulfonyl)imide, 99%; CB: carbon black; PEDOT:PSS: poly(3,4-ethylenedioxythiophene) polystyrene sulfonate; PDMS: polydimethylsiloxane; CNT = carbon nanotubes.

^a^All additives are expressed in weigh percent.

**Figure 1.**
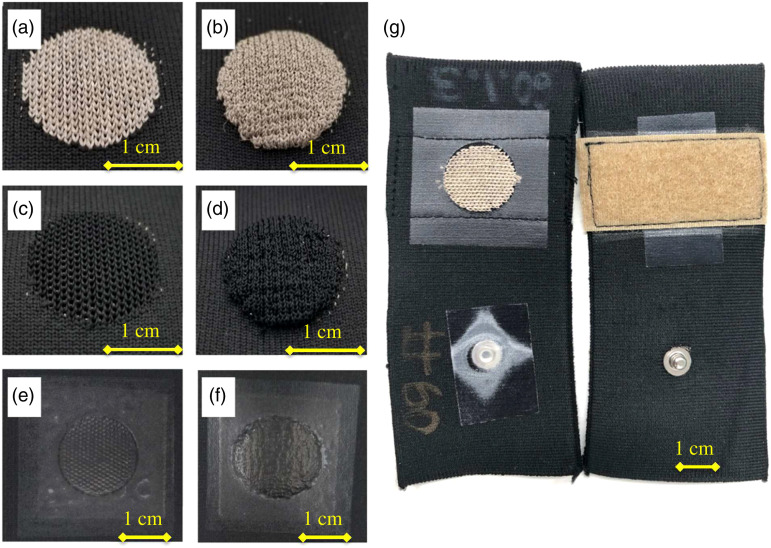
Textile electrode samples. (a) Flat silver, (b) raised silver, (c) flat carbon, (d) raised carbon, (e) flat screen-printed electrode, (f) raised screen-printed electrode, and (g) (Left)—view of circular electrode surface (d = 2 cm), laminated back of snap-on connector and (Right)—attachment velcro, male snap-on connector.

All sensors used in this study shared the same form factor, a circular (d = 2 cm) electrode was knit using a conductive yarn (silver or carbon, described above). The variations between samples are as follows: knit silver conductive yarn in flat (Figure 1(a)) and raised (Figure 1(b)) forms, knit conductive carbon yarn with a layer of silver yarn underneath in flat (Figure 1(c)) and raised (Figure 1(d)) forms, and samples with screen-printed coatings on top of flat (Figure 1(e)) and raised (Figure 1(f)) knitted silver and carbon electrodes. The textile electrodes were then linked via a hidden knitted silver line (l = 4 cm) to a snap-on connector (Romefast 0.156 series medical-electrical snap fastener, 12 stud) (Figure 1(g)). A non-conductive laminate was placed on the eye-let side of the snap-on connector to prevent electrical contact with the body that could distort the EMG signal. Velcro straps were sewn onto the opposite face of each sample to aid in consistent electrode placement and application of pressure (Figure 1(g)).

Polymer materials were screen-printed to create different material coatings. Screen printing was used as it is a simple and versatile technique well-suited for both the mass production of textiles and rapid prototyping.^45^ Descriptions and labels of the screen-printed coatings are shown in Table 2, with supplier information and properties in Table 3. To reduce variability during the manual screen-printing process, the same research assistant screen-printed all the coatings. All coatings were printed on the same day. Any coatings with visual irregularities or deformities were not used. While some of the coatings and their concentrations were off the shelf, the quantity of CB, and graphene added to the inks were determined during previous testing with the goals of improving conductivity, but also maintaining the ability to be dispersed in the ink solvent.

**Table 3.** Screen printing material supplier information and properties.

Material	Product name	Manufacturer	Properties
Carbon	PE671	DuPont, United States	Sheet resistivity: < 500 mΩsq/25μm Viscosity @ 25°C: 4075 PaS Solids @ 150°C: 30–34 %
Carbon black	CB STD	Cabot Corporation, United States	Viscosity: 1.71.9 g cm^−3^
PDMS + 1% CNT	SilQuan-C	NanoQuan, Canada	N.R.
Graphene	N006-P	Angstron Materials, United States	Solids content: ≥ 98.80%
XCMB resin^a^	XCMB - 7905	PPG Industries, Inc., United States	Solids content: 16.9% Viscosity: > 0.21 cm^2^/s
Ionic liquid^Bb^	IL-0023	IoLiTec, Germany	H_2_O content: ≤ 500 ppm
Neoprene^c^	Neoprene polychloroproprene	DuPont, United States	N.R.
Part B^d^	RTC 615B	Momentive Performance Materials, United States	Volume resistivity: 1.8 × 10^15^ Ωcm Viscosity @ 25°C: 4 PaS
PEDOT:PSS	EL-P5015	Agfa-Gevaert, Belgium	Sheet resistance: 190 Ω/sq Viscosity @ 25°C > 50 PaS Solids content: 2.5–5.5% wt
Anisotropic conductive thermoplastic adhesive^e^	EXP 2650-50	Creative Materials, United States	Volume resistivity: X and Y: 1 × 10^12^ Ωcm Z: 1 × 10^−4^ 0.0001 Ωcm

^a^Solvent for graphene.

^b^1-ethyl-3-methylimidazolium bis(triflouromethylsulfonyl)imide, 99%.

^c^Solvent for carbon black.

^d^Curing agent for PDMS, added in a 10:1 ratio for all PDMS-based inks.

^e^Material printed onto coatings to aid adhesion onto the textiles.

**Figure 2.**
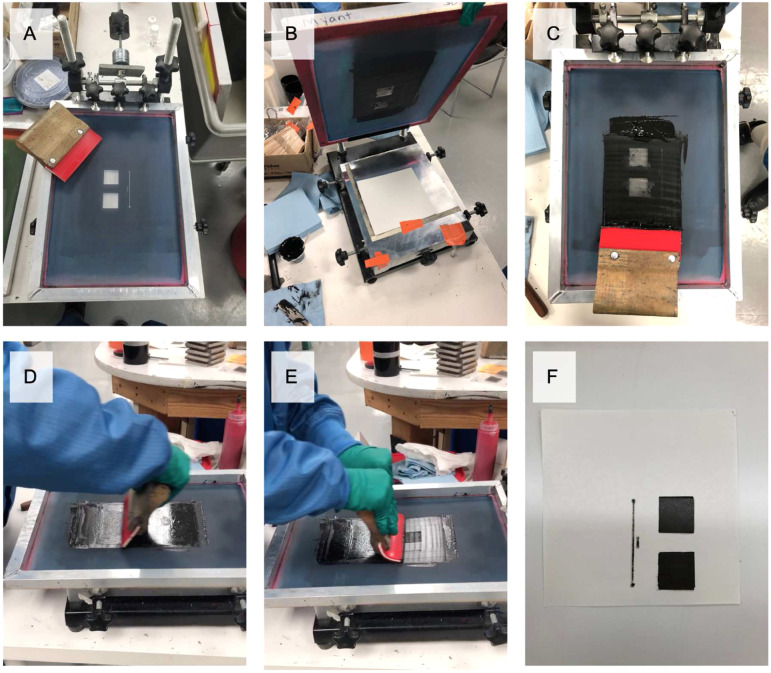
Screen printing setup and process. (a) Manual screen-printing setup, screen, and squeegee; (b) paper placement; (c) place ink on screen; (d) spread ink across screen; (e) push ink through screen; and (f) screen-printed electrode.

A manual screen printer was used to print the electrodes as 2.54 by 2.54 cm squares. Depending on the viscosity of the ink, either a 156- or 83-mesh count screen was used. The following method was used to screen-print coatings and transfer them to the electrodes:

1. If necessary, the inks were mixed. IL and Part B were manually mixed until visually homogeneous. CB and graphene inks were mixed using a 3 Roll Ink Mill (Torry Hills Technologies, United States).
2. A PDMS-finished heat transfer paper was placed beneath the screen. A squeegee was used to spread ink and then apply pressure on the screen, transferring the ink onto the paper (Figure 2). Note that coatings 9 and 10 were printed directly onto the textile, as contained PDMS and adhered too strongly to the paper.
3. After printing each layer, the print was cured according to the supplier recommendations. Layers were printed until a visually homogeneous surface (e.g., no gaps or bubbles) was formed.
4. For coatings 1 to 6, two layers of anisotropic conductive thermoplastic adhesive were printed after the coating to ensure conductive adhesion to the textile. Since PDMS adhered to the printing substrate, coatings 7 and 8 were printed on top of the thermoplastic adhesive (instead of under it), then manually peeled off the paper before step 5.
5. Using a heat press, coatings 1 to 8 were transferred at 250°C for 30 s onto the textile. During this time, the edges around the electrode surface were laminated with a transferable non-conductive polyurethane (Bemis Company, United States) to improve durability and ensure that the conductive contact area of each electrode was a circle 2 cm in diameter.

When referring to samples, the naming convention stated the knitted yarn, whether the knit was flat or raised, and the coating condition. For example, a sample named S-0-R refers to a silver (S) raised (R) electrode with no polymer coating (0), whereas S-0-F would be a silver (S) flat (F) electrode with no polymer coating (0). Similarly, C-5-F refers to a carbon (C) flat-knitted (F) electrode with a CB coating (5).

All coatings were tested on both flat-silver (S-#-F) and flat-carbon (C-#-F) electrodes. In the interest of feasibility and experiment time, only coatings 0 and 5 were tested on raised electrodes. Coating 0 was selected as it is the no-coating condition, giving a direct comparison of flat and raised structures. Coating 5 was selected as it was one of the best performing electrodes in preliminary testing.

### Impedance testing

A physical model of human skin was used to measure skinelectrode impedance, facilitating safe testing of the new materials and removing potential effects of variability in skin impedance seen across individuals. The model, designed to match test results on human skin,^46^ was constructed by dissolving 4.5% agar, 0.97% NaCl, and deionized water on a hot plate until boiling and then pouring the mixture into a glass container to cool and set. To measure impedance, an Ivium Potentiostat (Ivium Technologies, Netherlands) was used in 2- and 3-electrode configurations.^34^ A weight was placed on top of the electrodes to generate pressure (20 mmHg) between the electrode and agar, matching the maximum pressure at the electrodeskin interface during EMG collection. A recording was made after the measurement stabilized, ceasing to continually increase or decrease. Impedance for each sample was measured five times, for a total of 15 times per material. Measurements were performed at 100 Hz, targeting the peak frequency component for EMG.^47^

### EMG collection

**Figure 3.**
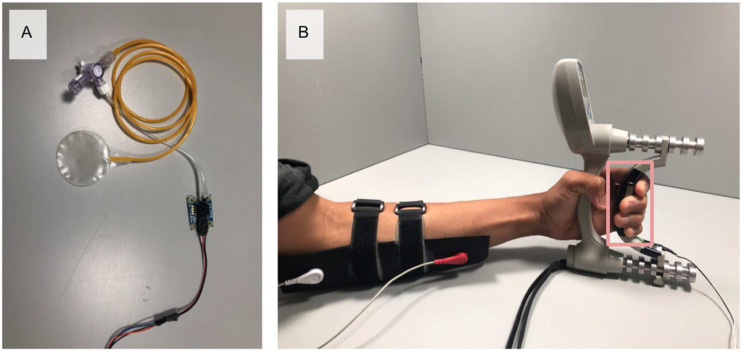
Experimental setup. (a) PicoPress sensor and transducer and (b) participant holding dynamometer and force sensor (highlighted) with electrodes on (the distance between the two straps is where the electrodes are contacting the body; the location of the female connectors correspond to the location of the male snap-on connector for each electrode).

EMG was collected from the dominant forearm of 10 adults during sub-maximal grip force measurements. The forearm was selected as it would allow for an easier quantification of contractions using a hand-based dynamometer (Figure 3). Testing on able-bodied participants was approved by the Research Ethics Board of the University Health Network (Toronto, Canada) under (REB16-6349.0). The order in which samples were tested was randomized for each participant to mitigate the effect of muscle. No skin preparation was performed. Two electrodes of the same size (d = 2 cm) were placed 2 cm apart (edge to edge or 4 cm center to center) on the participant’s dominant forearm over the flexor digitorum superficialis. Electrodes were centered around 1/4 of the distance between the medial epicondyle of the humerus and the distal sulcus carpi.^48^ A reference electrode of the same material was placed on the lateral epicondyle of the humerus on the non-dominant arm. To test the electrodes in conditions closest to their method of application, conventional skin preparation (i.e., remove of hair, abrasion of skin, and/or cleaning with alcohol wipes) was not used. While skin preparation is used to reduce impedance and improve signal quality in clinical applications, a wearable textile would not require skin preparation.

**Figure 4.**
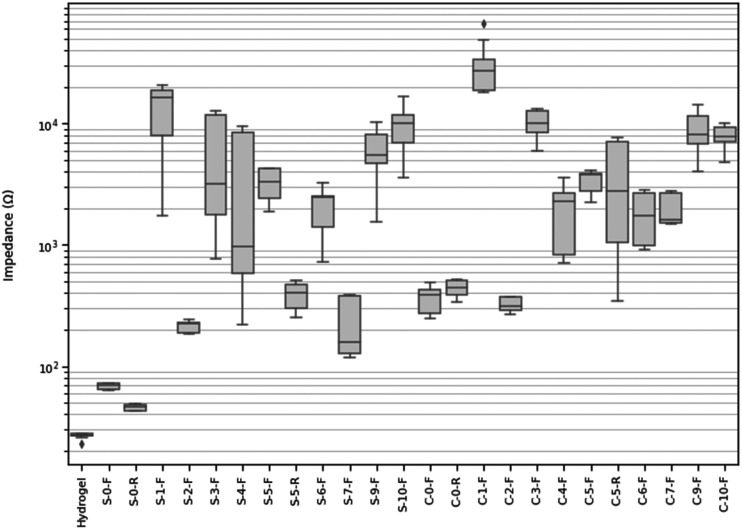
Mean impedance of the electrodes.

To control for pressure at the electrodeskin interface, a PicoPress (Microlab Elettronica, Italy) pneumatic pressure sensor was used. These are typically used to measure interface pressures of compression bandages.^49^ The PicoPress (Figure 4) was filled with 5 mL of air and connected to an Arduino Uno via a transducer (Absolute Air Pressure Sensor 1140, Phidgets, Canada), and then placed between the sensor band and skin. The band was adjusted until interface pressure was between 15 to 20 mmHg to provide a tight, but comfortable, fit and good signal quality.^8,14^ After fitting the band, the PicoPress was removed. While this lowered the pressure at the electrodeskin interface, participants still felt the band was tight, but comfortable. After validation of the skinelectrode interface pressure, signal acquisition was initiated. No settling time was allowed after application of electrodes or in-between the changing of electrodes, as this could lead to the development of perspiration, resulting in lower skinelectrode impedance and a higher signal-to-noise ratio.

A Jamar Plus+ Digital Hand Dynamometer (Jamar Technologies Inc., United States) with a 3.8 by 3.8 cm force-sensitive resistor (FSR 406, Interlink Electronics, United States) mounted on the handle; it was integrated with an Arduino Uno (Arduino, Italy) to measure and give visual feedback on contraction force during the experiments (Figure 3). Participants used the dynamometer to perform three isometric maximum voluntary contractions (MVC). During this, peak force was recorded from the dynamometer and the force sensitive resistor (FSR). This was repeated at approximately 25% and 50% MVC.

A custom biosensing acquisition board was used to collect EMG at 1000 Hz using unity gain active electrodes. The acquisition board contained a differential instrumentation amplifier followed by a first-order high-pass filter (Butterworth, f_c_ = 16 Hz) with unity gain and a third order low-pass Bessel filter (f_c_ = 500 Hz) with 3.56x gain. When collecting EMG, participants performed five contractions at 25% MVC, each 3 s long, with 10 s of rest in-between. This was repeated for contractions at 50% MVC. The resting portions between were used to calculate the baseline noise in the EMG signal. To guide contractions, the FSR values associated with 25% MVC and 50% MVC were displayed to participants along with a target force and indicators of when to relax and contract.

All post-processing was completed in MATLAB R2016b (MathWorks, United States). Using the force-sensitive resistor, the last 2.5 s of each contraction was isolated. Data collected during the rest periods between contractions was used to quantify noise. Motion artifact was removed from the EMG signal using a high-pass filter (eighth-order Butterworth, f_c_ = 5 Hz). After filtering, the following metrics were computed:

1. The mean and standard deviation of SNR^35^

$$SNR\;=RMS_{signal}/\;RMS_{noise}\;$$

2. For comparison between participants and across materials, SNRs were normalized by dividing by the SNR for the hydrogel electrode, to compute relative SNR (rSNR).

$$rSNR=SNR_{Sample}/SNR_{Hydrogel}$$

3. Power spectral density (PSD) was estimated using Welch’s PSD estimate. This was used to capture the full frequency distribution of each EMG signal.
4. Mean (MNF) and median (MDF) frequency were calculated using the built-in MATLAB functions to quantify the frequency distribution of the EMG signals.

Statistical analyses were performed in Rstudio (Rstudio, United States). To verify whether electrode material or contraction strength had an impact on normalized SNR, a repeated-measures analysis of variance (RMANOVA) was performed with sample material and contraction strength as within-subject factors. To identify which materials had significant differences in SNR, post-hoc pairwise two-sided t-tests were also used. To determine whether sample type significantly affected the frequency distribution, the correlation coefficient between the mean PSD for each material and the hydrogel electrodes was computed.

## Results

### Electrode preparation

All materials, except for coating 8, were fabricated without forming any visible cracks before and after transferring the coating to the electrode. When attempting transfer of coating 8, the polymer consistently cracked as it was being peeled off the substrate. As a result, coating eight did not advance to Phase 2.

### Impedance testing

**Figure 5.**
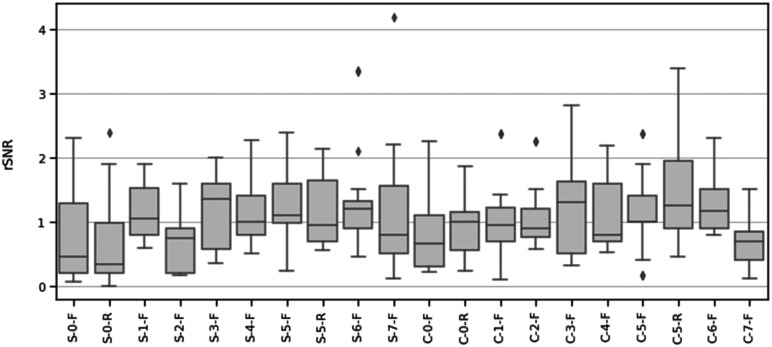
Mean relative signal-to-noise ratio (rSNR).

Figure 5 shows the mean impedance of each electrode material at 100 Hz. Based on the SENIAM recommendations, about 95% of EMG signal power is accounted for, along with harmonics at up to 400 Hz.^50^ Since the plateau in power of frequency in EMG recordings can be observed between 80–120 Hz, we selected 100 Hz from the impedance frequency sweep for comparison purposes against gel electrodes. For a more details, look into the range of frequencies assessed (1, 10, 100, 1 K, and 10 KHz) and please refer to our prior study.^44^ During impedance testing, coatings 9 and 10 were found to be abrasive and uncomfortable for the skin. As a result, these coatings did not advance to phase 3.

### EMG testing

**Figure 6.**
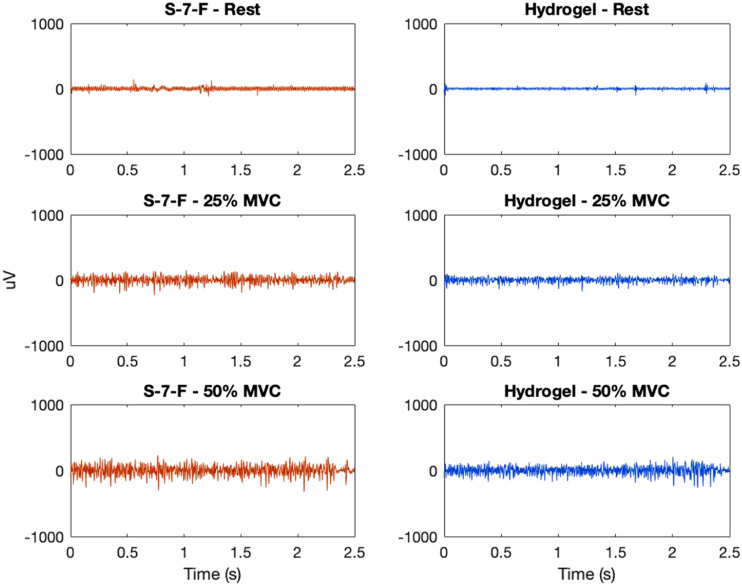
Comparison of electromyogram (EMG) at different contraction levels from electrode S-7-F (knitted silver, coating 7, raised) and hydrogel (H) for one participant—example of low relative signal-to-noise ratio (rSNR), rSNR_25%MVC_ = 0.50, rSNR_50%MVC_ = 0.51.

**Figure 7.**
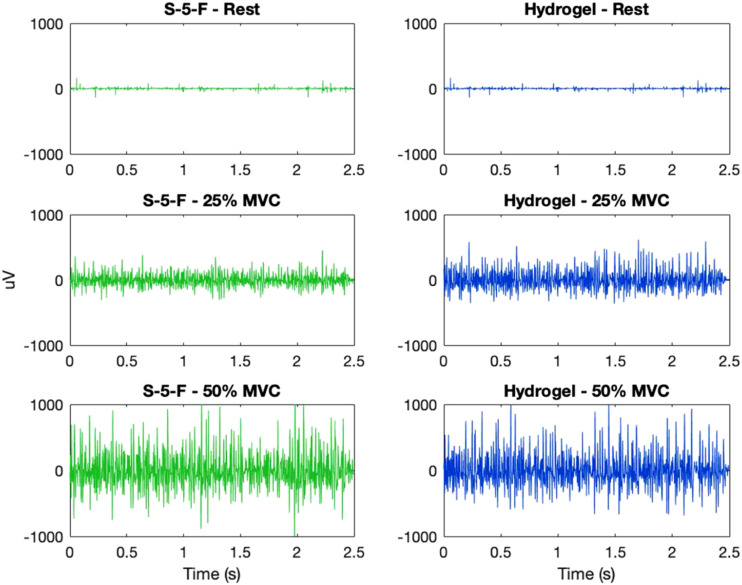
Comparison of electromyogram (EMG) at different contraction levels from electrode S-5-F (knitted silver, no coating, raised) and hydrogel (H) for one participant—example of high relative signal-to-noise-ratio (rSNR), rSNR_25%MVC_ = 1.95, and rSNR_50%MVC_ = 2.14.

EMG was collected from a total of 10 participants (6 male and 4 female) ages 20 to 26. Participants did not report any adverse reactions (i.e., skin redness, rash, and tenderness) during or after testing. The mean rSNRs for all materials across participants are summarized in Table 4 and displayed in Figure 5. Materials with a mean rSNR greater than one performed better than the hydrogel electrode, while those with mean rSNR less than one performed worse. The mean SNRs for hydrogel were SNR_25%MVC_ = 7.96 (8.08) and SNR_50%MVC_ = 13.66 (11.57), respectively. To illustrate low and high signal quality, the filtered EMG signals corresponding to the lowest and highest rSNR are shown in Figure 6 and Figure 7.

**Table 4.** Mean relative signal-to-noise ratio (rSNR) at different contraction levels.

Electrode	rSNR_25_	rSNR_50_	rSNR_all_
	Mean (SD)	Mean (SD)	Mean (SD)
*S-0-F*	0.78 (0.75)	0.66 (0.64)	0.72 (0.69)
*S-0-R*	0.72 (0.77)	0.57 (0.63)	0.64 (0.69)
*S-1-F*	1.19 (0.44)	1.02 (0.32)	1.10 (0.39)
*S-2-F*	0.72 (0.45)	0.64 (0.40)	0.67 (0.42)
*S-3-F*	1.17 (0.56)	1.13 (0.65)	1.15 (0.59)
*S-4-F*	1.16 (0.50)	1.25 (0.64)	1.21 (0.56)
*S-5-F*	1.28 (0.63)	1.26 (0.51)	1.27 (0.56)
*S-5-R*	1.19 (0.54)	1.17 (0.64)	1.18 (0.58)
*S-6-F*	1.39 (0.82)	1.34 (0.92)	1.36 (0.85)
*S-7-F*	1.03 (0.70)	1.20 (1.22)	1.12 (0.98)
*C-0-F*	0.89 (0.65)	0.85 (0.59)	0.88 (0.60)
*C-0-R*	0.97 (0.50)	0.90 (0.41)	0.93 (0.45)
*C-1-F*	1.08 (0.60)	0.97 (0.53)	1.02 (0.56)
*C-2-F*	1.09 (0.50)	1.05 (0.54)	1.07 (0.50)
*C-3-F*	1.24 (0.84)	1.13 (0.73)	1.19 (0.77)
*C-4-F*	1.14 (0.55)	1.17 (0.54)	1.15 (0.53)
*C-5-F*	1.19 (0.67)	1.15 (0.62)	1.17 (0.62)
*C-5-R*	1.54 (0.89)	1.34 (0.60)	1.44 (0.74)
*C-6-F*	1.31 (0.44)	1.24 (0.53)	1.28 (0.48)
*C-7-F*	0.74 (0.37)	0.63 (0.39)	0.69 (0.38)

**Figure 8.**
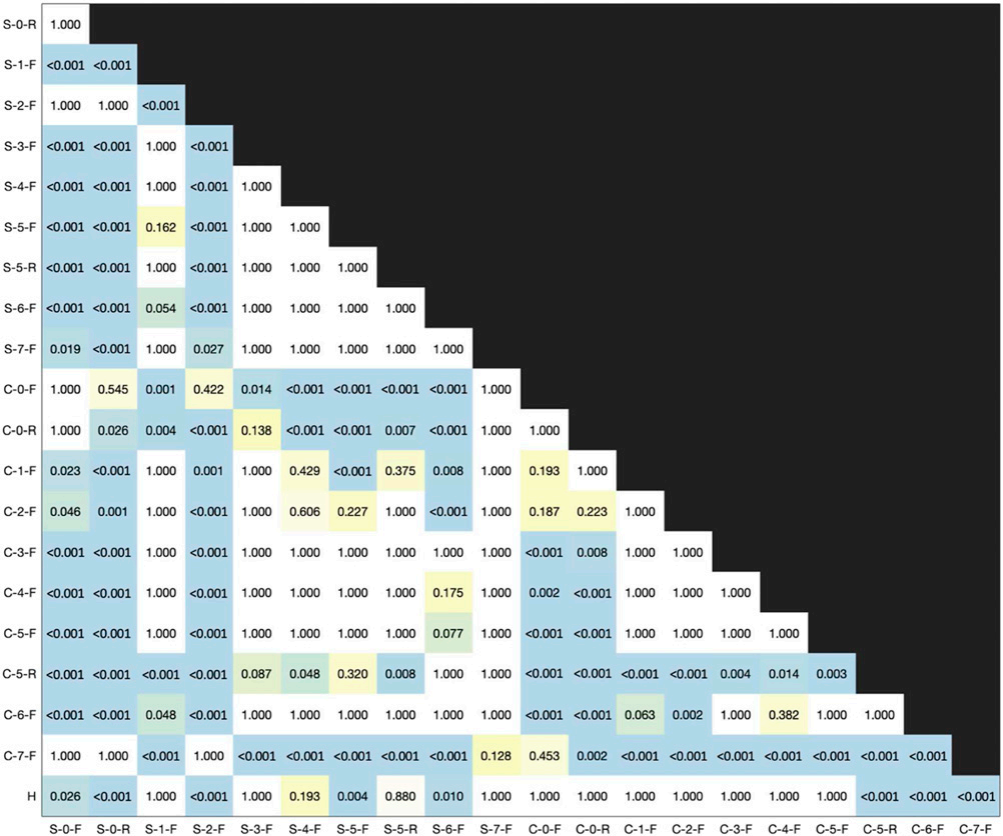
Post-hoc paired t-test results between mean relative SNR (rSNR) for all materials.

A RMANOVA for rSNR with material and contraction as within-subject variables revealed electrode material as a significant main effect (F = 2.714, *p* < 0.001, *η*^2^ = 0.206). Contraction strength (F = 1.160, *p* = 0.310, *η*^2^ = 0.004) and the interaction effect of material and contraction (F = 0.746, *p* = 0.774, *η*^2^ = 0.009) were not significant. Post-hoc, pairwise, t-tests were performed using a Bonferroni correction (Figure 8). A D’Agostino skewness test was used to verify the assumption of normality of the RMANOVA’s residuals (*p* = 0.236).

**Figure 9.**
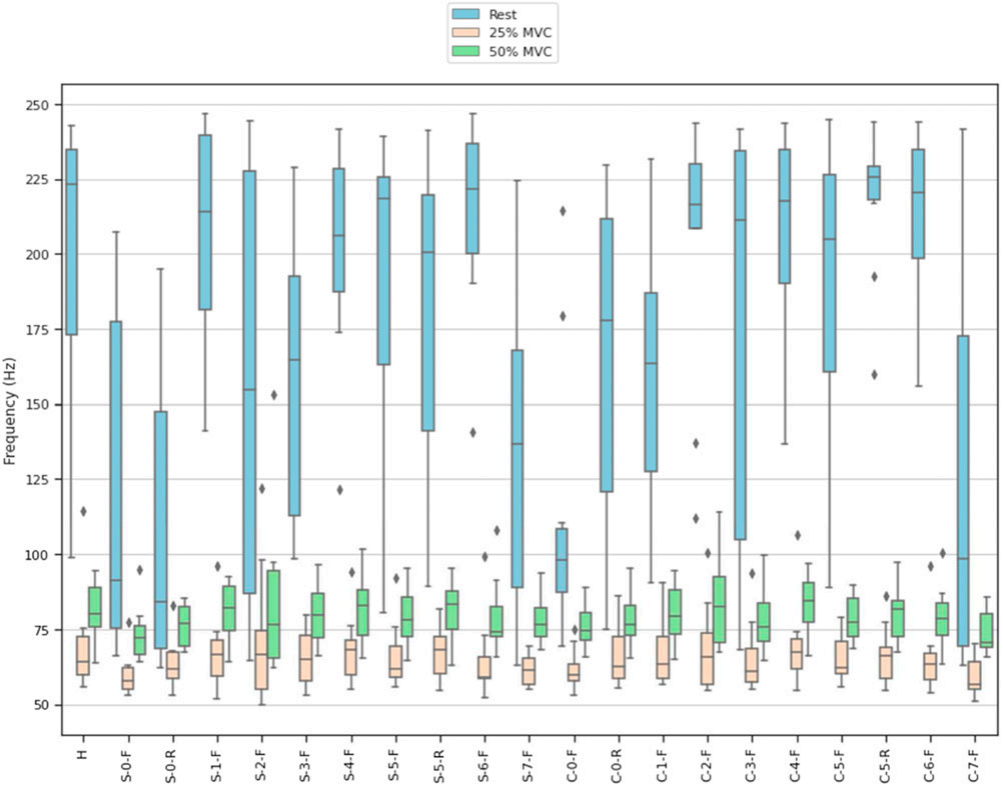
Mean frequencies (MNF) measured by electrodes.

**Figure 10.**
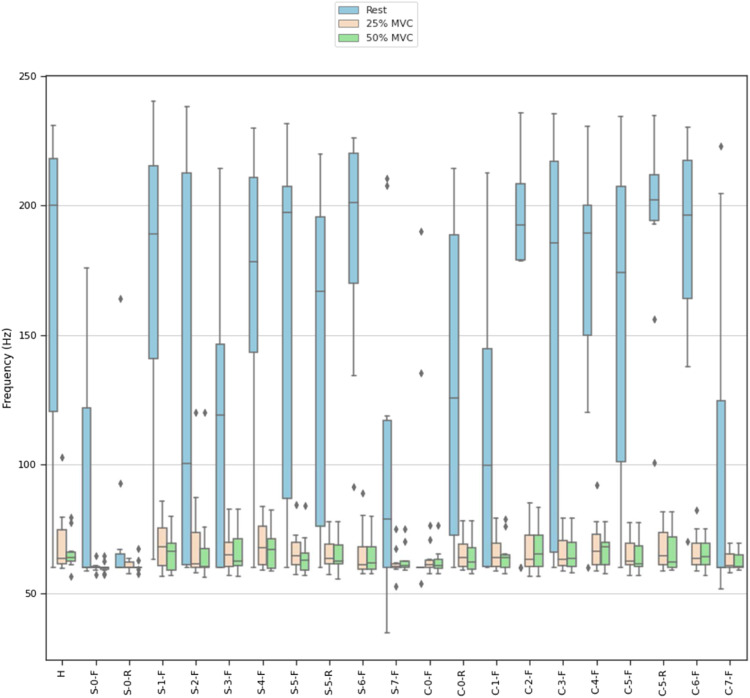
Median frequencies (MDF) measured by electrodes.

**Figure 11.**
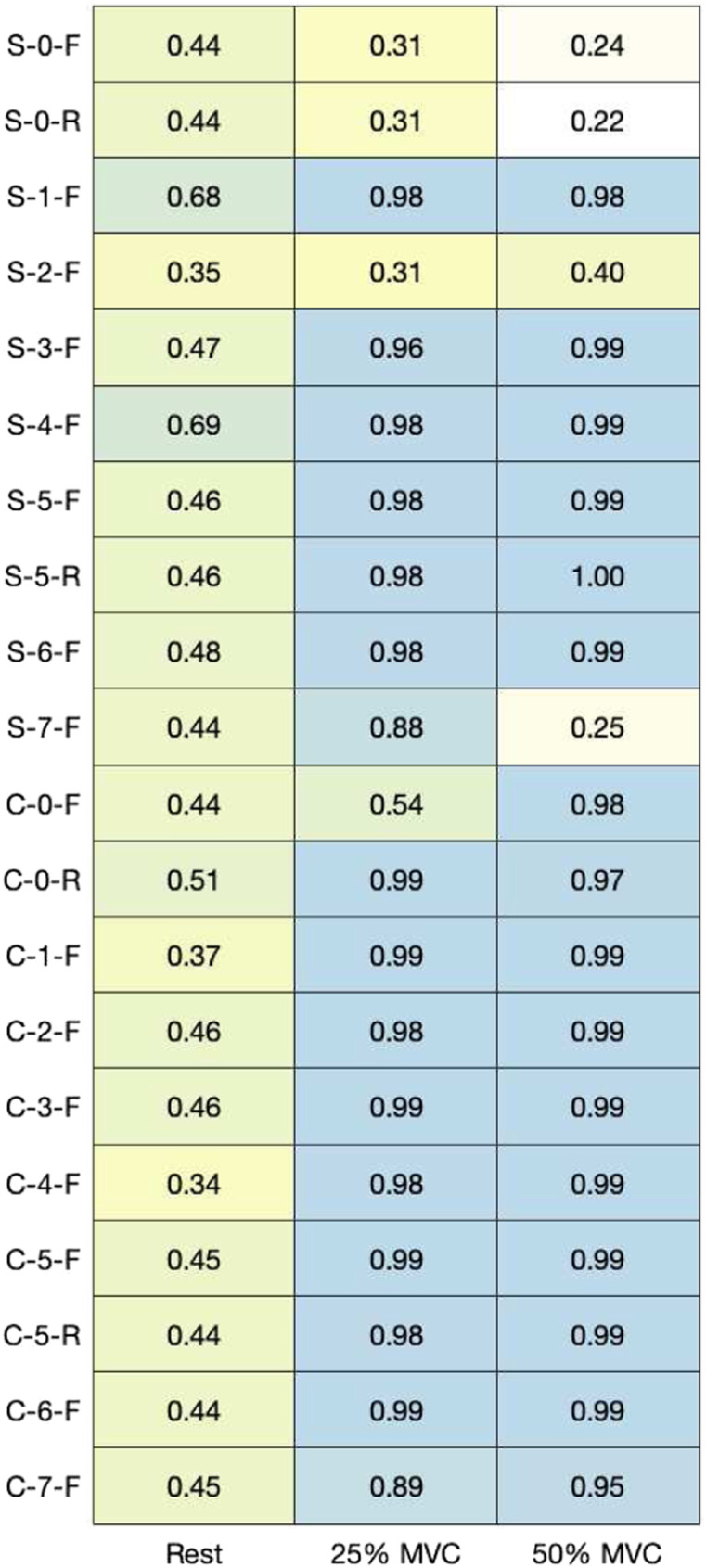
Correlation between power spectral densities for each material in comparison to hydrogel electrodes (*p* < 0.001).

**Figure 12.**
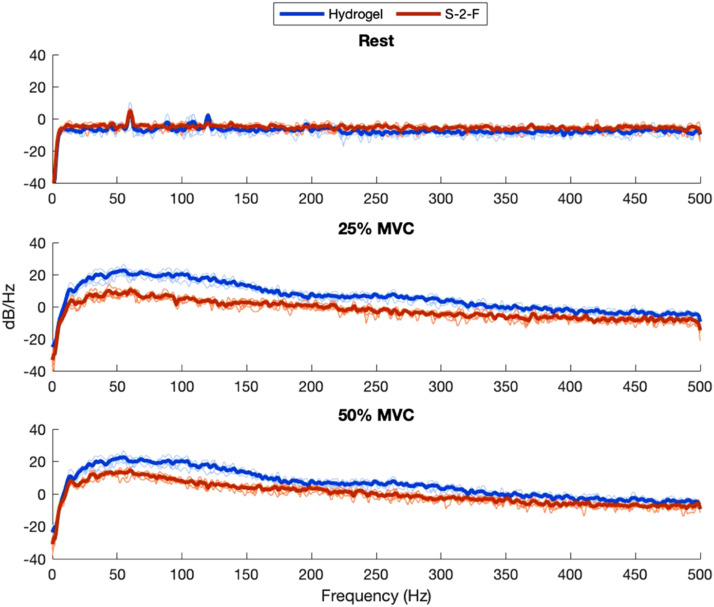
Example of low-power spectral density correlation (R^2^_Rest_ = 0.03, R^2^_25%MVC_ = 0.28, R^2^_50%MVC_ = 0.34).

**Figure 13.**
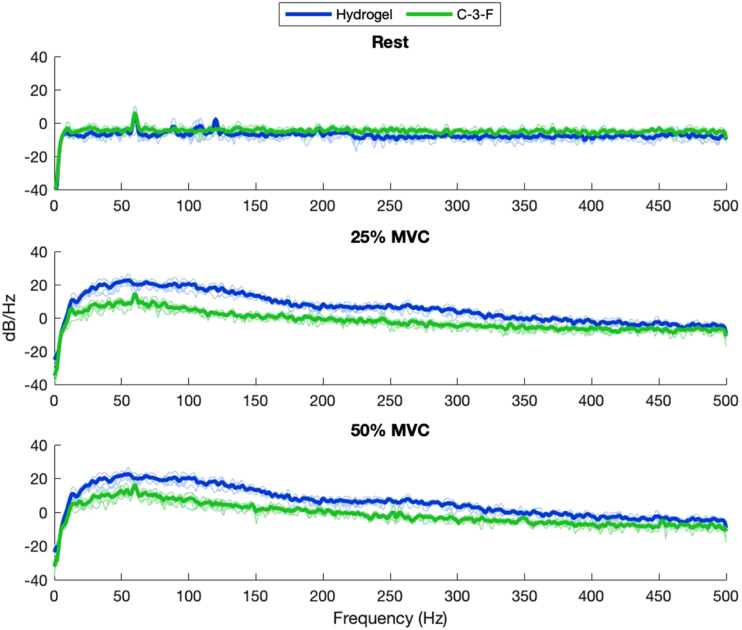
Example of high-power spectral density correlation (R^2^_Rest_ = 0.05, R^2^_25%MVC_ = 0.99, R^2^_50%MVC_ = 0.99).

MNF and MDF (Figures 9 and 10) were calculated across contractions to quantify the overall shape of the frequency spectrum captured by each electrode. Pearson’s correlation coefficients between the mean PSD for each material and the PSD for the hydrogel electrode were calculated (Figure 11). Figures 12 and 13 show examples of PSDs exhibiting a low and high correlation with the PSD of the hydrogel electrodes, respectively.

## Discussion

Based on the statistical analysis of the rSNR of each developed dry-contact electrode compared to gel electrodes, we determined that material selection played a significant role in determining signal quality. Conversely, the contraction strength was not found to be a determinant of signal quality when compared to gel electrodes. These findings demonstrated that material with an rSNR > 1 compared to gel electrodes can be used in applications where determinations with regard to the EMG signal is required for different contractions.

The materials were further categorized based on their rSNR to gel electrodes through post-hoc, pairwise t-tests, with the following three clusters forming:

Materials with higher rSNR than hydrogel (*p* < 0.05):

• SilverNeoprene + CB (7%)Flat (S-5-F)
• SilverNeoprene + CB (7%) + 5% ILFlat (S-6-F)
• CarbonNeoprene + CB (7%)Raised (C-5-R)
• CarbonNeoprene + CB (7%) + 5% ILFlat (C-6-F)

Materials with rSNR similar to hydrogel:

• SilverCarbonFlat (S-1-F)
• SilverXCMB + 10% GrapheneFlat (S-3-F)
• SilverXCMB + 10% Graphene + 5% ILFlat (S-4-F)
• SilverNeoprene + CB (7%)Raised (S-5-R)
• SilverNeoprene + CB (7%) + 5% ILFlat (S-6-F)
• SilverPEDOT:PSS +12.5% (PDMS + 1% CNT)Flat (S-7-F)
• CarbonNoneFlat (C-0-F)
• CarbonNoneRaised (C-0-R)
• CarbonCarbonFlat (C-1-F)
• CarbonCarbon +5% ILFlat (C-2-F)
• CarbonPEDOT:PSS +12.5% (PDMS + 1% CNT)Flat (C-7-F)

Materials with lower rSNR than hydrogel (*p* < 0.05):

• SilverNoneFlat (S-0-F)
• SilverNoneRaised (S-0-R)
• SilverCarbon +5% ILFlat (S-2-F)
• CarbonPEDOT:PSS +12.5% (PDMS + 1% CNT)Flat (C-7-F)

Noting that electrodes of the same material could have a wide range of impedances (Figure 4), leading to impedance mismatch, and lower signal quality, the correlation between the coefficient of variance of impedance (COV_Z_) and rSNR was examined (R^2^= 0.184, *p* < 0.001). The positive correlation suggests that, in this study, materials with higher COV_Z_ had a higher rSNR. As such, impedance mismatch is not a reason that some materials had a lower signal quality than others.^15,19,20^

When examining the combinations of materials (yarns, additives, and form factor) used, there are no obvious patterns as to which individual materials result in higher signal quality. Half of the electrodes with significantly higher rSNR than hydrogel used silver yarn, while the other half used carbon. However, silver yarn had three electrodes that performed significantly worse than hydrogel, whereas carbon yarn only had one. When comparing the same coating condition on the silver versus carbon yarn, 5/8 coatings had a higher rSNR on silver yarn, with none of these differences reaching significance. 3/8 coatings had higher rSNR on carbon yarn, with the difference reaching significance for coating 2 (carbon and 5% IL, *p* < 0.001). The effect of raised electrodes was unclear, with most of the rSNR values being very similar. However, CarbonNeoprene + CB (7%)Raised (C-5-R) had a higher rSNR than CarbonNeoprene + CB (7%)Flat (C-5-F), with the difference reaching significance (C-5-R: rSNR_all_ = 1.44 (0.74), C-5-F: rSNR_all_ = 1.17 (0.62), *p* = 0.003). While, adding IL improved rSNR for 4/6 coatings, none of the differences were significant. The addition of IL to coating 1 significantly lowered signal quality (SilverCarbonFlat (S-1-F): rSNR_all_ = 1.10 (0.39), SilverCarbon +5% ILFlat (S-2-F): rSNR_all_ = 0.67 (0.42), *p* < 0.001).

The MNF, MDF, and PSD (Figures 9–11) for each electrode indicate that the frequency component captured by most of the electrodes is similar to that of hydrogel. While no materials had strong PSD when at rest, 15/20 electrodes exhibited PSD correlations greater than 0.9 at 25% and 50% MVC. Considering that the aim of this study was the assessment of EMG signals from different materials compared to conventional gel electrodes, the frequency component during contractions was of primary interest. Therefore, it was deemed acceptable that the frequency component of the electrodes differs at rest, when compared to gel electrodes. A notable reason for the difference in the frequency component at rest was that the textile-based electrodes seem to have a higher baseline noise than the hydrogel electrode. Since SilverNoneFlat (S-0-F), SilverNoneRaised (S-0-R), SilverCarbon + 5% ILFlat (S-2-F), SilverPEDOT:PSS + 12.5% (PDMS + 1% CNT)Flat (S-7-F), and CarbonNoneFlat (C-0-F) exhibited weak PSD correlations under several conditions, these materials may not be the best candidates for textile-based electrodes.

After considering SNR and the frequency spectrum of signals collected by the electrodes, the most successful electrodes in this study were SilverNeoprene + CB (7%)Flat (S-5-F), SilverNeoprene + CB (7%) + 5% ILFlat (S-6-F), CarbonNeoprene + CB (7%)Raised (C-5-R), CarbonNeoprene + CB (7%) + 5% ILFlat (C-6-F). All of these electrodes were based on the coating formulation of neoprene and 7% CB, with or without the addition of 5% IL. These materials had significantly higher rSNRs than hydrogel electrodes without distorting the frequency composition of the EMG signal. The signal quality resulting from flexible CB electrodes has also been observed by Reyes et al. who constructed CB/PDMS electrodes for underwater ECG and EMG measurement, showing similar qualitative results between the hydrogel and CB electrodes.^27–29^

### Limitations

While this study examines a wide array of materials found in the literature, the processes used in each paper and material compositions were not replicated exactly. A uniform process was employed to help standardize implementation; similarly, the primary constituents of the materials were selected to isolate the plurality of additives. Some of the tested inks were made or modified in-house, whereas other inks were purchased and used as is. For purchased components, the exact materials compositions were not provided by the suppliers and as such, remain unknown. For inks modified in-house, the mixing process could lead to variability in ink coating composition, leading to variability in EMG and impedance measurements. The manual nature of screen printing could have also introduced variability into sensor thickness and consequently, EMG and impedance measurements. This could be improved, by using automated screen-printing equipment, whose parameters can be controlled. While variability in sensor construction is reflected in the standard deviations of impedance measurements for some of the materials, the positive correlation between COV_Z_ and rSNR suggests that impedance mismatch may not have been a substantial factor in determining signal quality. Additionally, this study examined a vast number of materials with a relatively small sample size. While efforts were made to curb this by having participants perform several contractions for each material and using a within-subject approach to statistical analysis, the study may be underpowered to find significant differences between some materials. Given this, our study provides a base comparison for a wide variety of electrode materials, allowing the identification of promising materials. The differences and potential advantages of certain materials are worth further investigation in a larger study with a greater sample size, using fewer electrode material variations.

An important consideration for skin contactbased electrodes will be the biocompatibility and cytotoxicity of the materials. Skin reactions such as contact dermatitis, pruritus, hypo-/hyper-pigmentation, and erythema have been well reported following the application of conventional hydrogel based adhesive electrodes. These reactions can be acute in nature after a temporary application (i.e., < 1 h clinic visit recording) or more pronounced with long-term continuous application (i.e., > 24 h Holter monitor). These have been attributed to the components in the conductive gel, as well as the constituents of the adhesive surrounding the electrodes. In the case of dry-contactbased electrodes, these components are not present, though for the new set of materials, the dermal affects need to be observed and studied. Standards such as

• ISO 10993: biological evaluation of medical devices based on the nature and duration of their contact with the body, part 10, testing for irritation and skin sensitization;
• OECD 404: acute dermal irritation/corrosion, assessing health hazards from exposure to solid substances by dermal application; and
• OECD 406: skin sensitization from exposure to test substances via epidermal application,

can be followed and tested against. Performing these tests was outside of the scope and budget of this study due to the broad nature of materials being examined. As a subsequent step, after the narrowing and selection of materials that best suit the use case, performing these tests would be suggested. They would validate the efficacy of each material for short- and long-term dermal applications.

Motion artifact is a common factor that is observable in electrophysiological signal acquisition during ambulatory conditions. In EMG use cases such as rehabilitation, athletics, or remote patient monitoring, having the ability to capture clean EMG signals with high enough SNR, despite the presence of these artifacts, is critical for sensible interpretations. For the scope of this study, we aimed to select the optimal material that provided the best relative SNR and PSD compared to gel electrodes for EMG signals of the forearm. To accurately quantify these metrics and limit the impact of motion artifacts on measurements, the forearm readings were done in a stationary state. From the derived best-performing materials, a follow-up study should be performed to capture and quantify the effect of motion artifact during ambulatory conditions. This will allow for further refinement in the selection of the best performing materials. In such a study, the pressure applied to the electrodes will be an important variable to account for and test against. Numerous studies have assessed the effect of skinelectrode pressure, with 15–20 mmHg appearing to be an optimal range to consider and start with.^8,14^ As these electrodes will be used in textile form factor developing toward a wearable, the ability to create conformal and homogenous pressure to the applied limb or body segment is an important design consideration. Another important variable to account for is the taction or stickiness of the selected material to skin. Since these dry-contact electrodes are missing the adhesive component of gel electrodes, there is a likelihood that they would have shear forces changing their position across the skin, creating motion artifact components, affecting readings. Quantifying taction in a planar direction and assessing its effect in tandem with the applied pressure from the garment would lead to a comprehensive understanding of the optimal material-form factor design requirements for EMG-based wearables.

## Conclusion

This study looked to gain an understanding of how different biopotential electrode materials discussed in the literature compared against each other to provide context and continuity to existing research. In this study, a wide variety of materials used for textile biopotential measurement were surveyed and tested while controlling for factors known to affect signal quality, such as interface pressure and contraction level. It was found that neoprene and 7% CB coatings on knitted silver and carbon electrodes performed better than hydrogel electrodes when measuring EMG. It is worth investigating the use of CB and neoprene further, by testing it with a larger sample size and for other biopotential monitoring applications, such as ECG and EEG. Future studies in biopotential material development should investigate washability tests to understand the long-term stability of the screen-printed CB and neoprene coating as well as finding a way to transform the coatings into yarns that can be more easily produced and commercialized.
